# The Impact and Burden of Dietary Sugars on the Liver

**DOI:** 10.1097/HC9.0000000000000297

**Published:** 2023-11-06

**Authors:** Helaina E. Huneault, Ana Ramirez Tovar, Cristian Sanchez-Torres, Jean A. Welsh, Miriam B. Vos

**Affiliations:** 1 Nutrition and Health Sciences Program, Laney Graduate School, Emory University, Atlanta, Georgia, USA; 2 Division of Gastroenterology, Hepatology, and Nutrition, Department of Pediatrics, Emory University, Atlanta, Georgia, USA

## Abstract

NAFLD, or metabolic dysfunction–associated steatotic liver disease, has increased in prevalence hand in hand with the rise in obesity and increased free sugars in the food supply. The causes of NAFLD are genetic in origin combined with environmental drivers of the disease phenotype. Dietary intake of added sugars has been shown to have a major role in the phenotypic onset and progression of the disease. Simple sugars are key drivers of steatosis, likely through fueling de novo lipogenesis, the conversion of excess carbohydrates into fatty acids, but also appear to upregulate lipogenic metabolism and trigger hyperinsulinemia, another driver. NAFLD carries a clinical burden as it is associated with obesity, type 2 diabetes, metabolic syndrome, and cardiovascular disease. Patient quality of life is also impacted, and there is an enormous economic burden due to healthcare use, which is likely to increase in the coming years. This review aims to discuss the role of dietary sugar in NAFLD pathogenesis, the health and economic burden, and the promising potential of sugar reduction to improve health outcomes for patients with this chronic liver disease.

## INTRODUCTION

NAFLD, or metabolic dysfunction–associated steatotic liver disease,^1^ was initially described in the early 1800s.^2^ Since then, NAFLD has grown from a relatively unknown disease to a major cause of liver-related morbidity and mortality.^3^ NAFLD is clinically characterized by steatosis occupying > 5% of hepatocytes in the absence of alcohol consumption.^4,5^ The condition exists on a spectrum that ranges from simple steatosis to NASH, or metabolic dysfunction-associated steatohepatitis,^1^ which can progress to fibrosis and cirrhosis, predisposing patients to HCC.^6^ NAFLD is closely associated with other metabolic disorders such as type 2 diabetes (T2D), metabolic syndrome, polycystic ovarian syndrome, obesity, dyslipidemia, and cardiovascular disease (CVD).^7–9^

### Population trends

In association with the obesity epidemic and the major changes in the food supply over the past few decades, the global prevalence of NAFLD has risen markedly to an estimated 25.2%. By 2040, it is projected that over half the adult population will have NAFLD.^10^ In the United States, the prevalence has risen to ≥ 34.0% in adults,^11^ and 11% in adolescents, ranging from 27% to 43% of those with childhood obesity.^5^ There are marked differences in NAFLD prevalence by nation, ethnicity, and race within countries. These differences are largely due to socioeconomic factors, sugar consumption, and, in part, due to the population prevalence of genetic polymorphisms underlying the susceptibility for developing NAFLD.^12,13^ In the United States, the highest prevalence of NAFLD is seen in those of Hispanic heritage, possibly driven by the inheritance of the most prevalent polymorphism associated with NAFLD, patatin-like phospholipase domain–containing protein three (PNPLA3).^14^

### Healthcare burden

NAFLD and its complications cause a considerable healthcare burden worldwide. According to Younossi et al,^15^ the US annual direct medical costs of NAFLD are ~$103 billion, and in the European countries (Germany, France, Italy, and the United Kingdom), the annual cost is about $35 billion, with total costs highest in patients aged 45–65.^15^ Furthermore, in the US, the annual hospitalization rate due to NAFLD has tripled from 2007 to 2014, with a greater increase in males versus females and Latino/Hispanics versus other ethnicities.^16^ Recent data suggest that NAFLD will become the most common indication for liver transplantation in the near future.^17^ NAFLD accounts for the highest increase in disability-adjusted life years compared to other liver-related chronic diseases.^18^ At the individual level, the cost of medical care (due to testing, monitoring, and hospitalization) for a patient with NAFLD is estimated to be nearly twice as high compared with healthy individuals.^15^ In addition, there is an indirect societal impact due to absenteeism, caregiver burden, and reduced health-related quality of life.^19^

## THE ROLE OF SUGAR IN NAFLD PATHOPHYSIOLOGY

**FIGURE 1:**
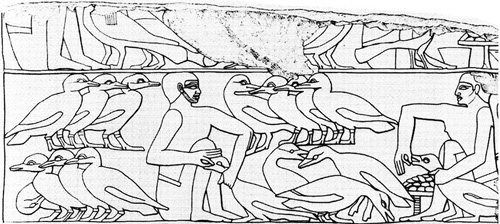
A bas relief depiction from the tomb of Mereruka, 2500 BC, illustrating the ancient Egyptian practice of overfeeding geese to produce *foie gras*.^20^

While the causes of NAFLD are multifactorial, dietary intake of added sugars, especially fructose, has been of interest as a driver of the disease for a long time.^20^ The link between steatosis in the liver and fructose consumption has been attributed to Pliny, the Elder, who wrote that Marcus Apicius, the famous Roman chef, would make fatty liver (*foie gras*) by overfeeding geese dried figs (a rich source of fructose). The process of force-feeding geese originally comes from ancient Egypt, with depictions of the practice found in the tomb of Mereruka dated 2500 BC^21^ (Figure 1). Furthermore, in 1860, the German chemist Justus von Liebig also observed that dietary carbohydrates stimulated steatosis in the liver.^13,22^

Excessive dietary sugar intake is thought to hold a major role in the onset and progression of NAFLD. The Dietary Guidelines for Americans 2020–2025 and the World Health Organization recommend that the intake of added sugars should not exceed 10% of total energy intake.^23,24^ This translates to ~50 gm (200 calories) of sugar on a 2000-calorie/day diet. A further reduction to less than 5% of total energy intake is recommended for additional health benefits.^25^ While total sugar consumption in the United States has decreased over recent years,^26^ current intakes remain above these guidelines, with sugar-sweetened beverages (SSB) as the top source, followed by desserts and sweet snacks.^27^ The average American adult and child consume about 17 teaspoons (68 gm) of added sugar per day,^28^ and the most common added sugars in the contemporary human diet include sucrose (table sugar) and high fructose corn syrup.^29^

**FIGURE 2:**
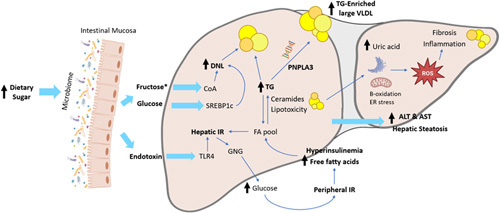
Biological mechanisms of NAFLD development by a high free sugar diet. Dietary sugars in the gut can alter the microbiome, increasing endotoxin that promotes hepatic IR.^30^ Monosaccharides enter the liver, and fructose is metabolized into fructose-1-P and further into acetyl-CoA, fueling DNL.^12^ Fructose, glucose, and insulin activate ChREBP & SREBP-1c, which transcriptionally activates genes in DNL. FA accumulation can exceed the liver’s capacity, which leads to ectopic lipid deposition and lipotoxicity. This promotes impaired mitochondrial beta-oxidation and ER stress, driving the production of ROS, hepatic IR, inflammation, and fibrosis through a complex set of mechanisms.^31^ Fructose metabolism also produces a drop in intracellular phosphate, resulting in increased uric acid formation, which is associated with oxidative stress and hepatic fat accumulation.^32^ Abbreviations: ALT, alanine aminotransferase; AST, aspartate aminotransferase; ChREBP, carbohydrate-responsive element–binding protein; DNL, de novo lipogenesis; ER, endoplasmic reticulum; FA, fatty acid; GNG, gluconeogenesis; IR, insulin resistance; PNPLA3, patatin-like phospholipase domain–containing protein 3; ROS, reactive oxygen species; SREBP-1c, sterol regulatory element–binding protein 1c; TG, triglycerides; TLR4, toll-like receptor 4. Figure adapted with permission from Welsh et al., 2023.

Recent evidence suggests that the overconsumption of added dietary sugars, especially fructose, precipitates hepatic steatosis due to complex mechanisms that ultimately promote increased lipogeneses and impaired fatty acid oxidation^13^ (Figure 2). Conversely, reducing the consumption of free sugars (added sugars and naturally occurring sugar in fruit juice, honey, etc.) can significantly improve hepatic steatosis in both adults and children with NAFLD.^33–35^

### Sugar and digestive mechanisms

It is important to note that although fructose and glucose share the same molecular formula (C_6_H_12_O_6_), they are absorbed and metabolized differently in the gut.^36^ Glucose is transported from the intestinal lumen into the enterocytes through an energy-requiring process mediated by the sodium-glucose cotransporter 1, whereas fructose is absorbed through a facilitated passive transport mechanism by GLUT5 on the apical border of enterocytes.^37,38^ At the basolateral membrane, GLUT2 facilitates the passive absorption of both glucose and fructose into the circulation.^36^ The small intestine plays a key role in dietary fructose metabolism. Studies in mice have shown that within the enterocyte, the majority of fructose is converted to glucose and other organic acids. Thus, the small intestine acts as a shield, protecting the liver from the lipogenic effects of fructose. However, excessive fructose intake can overwhelm intestinal fructose absorption, which is transported to the liver through the portal vein.^36,39^ A recent study measured fructose absorption/metabolism in 9 children with biopsy-proven NAFLD compared with 6 obese and 9 lean non-NAFLD controls, ages 8–18 years. The subjects with NAFLD demonstrated increased absorption and exaggerated metabolic response (elevated serum glucose, insulin, and uric acid) to fructose administration compared to lean children, while obese children without NAFLD had an intermediate response.^40^

### Sugars and gut microbial mechanisms

The human gut microbiome plays a significant role in human health and disease.^41^ The liver is directly linked to the intestines through the portal vein and is a major site for the detoxification of products coming through the portal blood, including microbial metabolites.^42^ In the digestive tract, excessive dietary sugars can alter resident microbial diversity, promoting dysbiosis, increased gut permeability,^43^ and elevated circulating endotoxin (lipopolysaccharide).^30^ In both adults and children with NAFLD, plasma endotoxin levels are elevated, suggesting either a failure in endotoxin removal or an increase in production that surpasses the liver's cleanup mechanisms.^30,44^

In healthy physiology, endotoxins circulate in the bloodstream at low concentrations,^45^ and the majority are cleared by the liver (~80%).^46,47^ Although the mechanisms are still being investigated, recent evidence in animal models revealed that LPS disappears rapidly from the circulation (half-life of 2–4 min) and is scavenged primarily by the liver sinusoidal endothelial cells, which possess a high endocytic ability, and to a lesser extent by the Kupffer cells.^48^ In patients with NAFLD, the function of the Kupffer cells is impaired, which may result in disturbed hepatic clearance and increased levels of circulating LPS, leading to accelerated liver injury.^49^ Endotoxemia activates the innate immune system and stimulates hepatic toll-like receptor 4, which induces inflammasomes and proinflammatory cytokines, promoting hepatic inflammation and insulin resistance (IR) (Figure 2).^50,51^ In children with NAFLD, administration of high fructose beverages caused postprandial rises in endotoxin.^44^ Studies in animal models have shown that high-sugar diets increase the relative abundance of LPS-producing *Proteobacteria* in the gut while simultaneously decreasing the abundance of *Bacteroidetes spp.*, some of which are considered protective against the effects of endotoxin.^52–54^ A recent human study revealed that a diet supplemented with high-fructose syrup significantly altered microbial composition, notably reducing the abundance of the genus Ruminococcus, known for its beneficial butyrate-producing bacteria.^55^ Furthermore, high doses of dietary fructose can overwhelm intestinal fructose absorption and clearance, causing fructose to spill over to the colon.^36,39^ In the colon, fructose generates the short-chain fatty acid acetate through microbial fermentation.^56^ Acetate can enter the portal circulation and be converted to acetyl-CoA by acetyl-CoA synthetase in the liver, potentially providing more carbon sources for de novo lipogenesis.^42^

### Sugars and hepatic mechanisms

**FIGURE 3:**
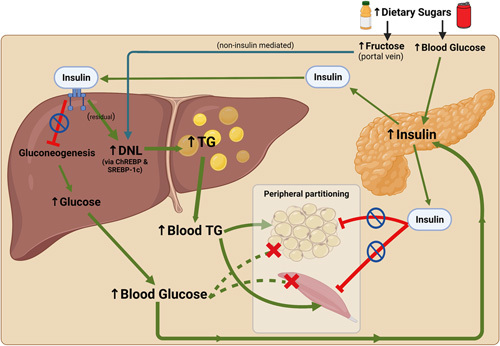
Mediators of hepatic de novo lipogenesis in obese individuals with NAFLD & insulin resistance. Dietary glucose stimulates insulin secretion from pancreatic beta cells. With hepatic IR, insulin fails to suppress hepatic gluconeogenesis; however, stimulation of DNL continues through residual activation of SREBP-1c. Dietary fructose is transported through the hepatic portal vein directly to the liver, where it stimulates de novo lipogenesis through non-insulin–mediated activation of ChREBP. Additionally, IR in adipose and muscle tissue prevents peripheral glucose uptake. Overall, these processes promote hyperglycemia and hyperlipidemia, driving NAFLD progression.^65–67^ Abbreviations: ChREBP, carbohydrate-responsive element–binding protein; DNL, de novo lipogenesis; IR, insulin resistance; SREBP-1c, sterol regulatory element–binding transcription factor 1; TG, triglycerides. Figure created using Biorender.

At the center of NAFLD pathogenesis, there exists an imbalance between hepatic lipid accumulation and removal, which is driven by inappropriately increased de novo lipogenesis. In hepatocytes, the monosaccharides glucose and fructose drive de novo lipogenesis through transcriptional activation of sterol regulatory element–binding protein-1c (SREBP-1c) and carbohydrate-responsive element–binding protein (ChREBP), which results in increased hepatic steatosis and directly affects insulin signaling and lipotoxicity.^31,57^ These monosaccharides also directly fuel de novo lipogenesis by providing necessary substrates for fatty acid and triglyceride synthesis, including acetyl-CoA and glycerol.^58,59^ Tracer experiments have shown that the contribution of de novo lipogenesis to intrahepatic lipid stores in NAFLD patients is ~26%, with ~59% derived from circulating fatty acids and ~15% from dietary fats.^60^ Additionally, a study comparing patients with NAFLD to healthy controls without steatosis revealed that de novo lipogenesis was 3-fold greater in NAFLD subjects.^61^ Interestingly, in individuals with obesity *and* NAFLD, Smith et al found a much larger contribution of de novo lipogenesis (~40%) to intrahepatic triglyceride formation, suggesting that elevated de novo lipogenesis is a distinct feature of NAFLD pathophysiology.^62^ They also demonstrated that increases in circulating insulin stimulate hepatic de novo lipogenesis in individuals with NAFLD.^62^ Further, they explored the concept that insulin mechanisms in the liver diverge, whereas the insulin receptors for glucose metabolism are resistant, leading to increased glycemia; the lipogenic mechanisms are intact and are overstimulated by excess insulin action lipogenesis.^62–64^ On the other hand, a study by Ter Horst et al revealed that patients with NAFLD and ce novo lipogenesis due to increased availability of lipogenic substrates like dietary fructose, while glucose-stimulated/insulin action lipogenesis was attenuated. However, the authors also stated that residual insulin-driven expression of lipogenic enzymes through SREBP-1c activation likely still plays a role. (Figure 3).^65^ Furthermore, insulin-resistant livers fail to suppress the activation of the transcription factor FoxO1, which upregulates 2 genes required for gluconeogenesis, phosphoenolpyruvate carboxykinase and glucose 6-phosphatase, leading to hyperglycemia.^66^

A recent study of 40 adolescent boys aged 11–16 years with NAFLD, undergoing an 8-week feeding protocol, demonstrated that the rate of de novo lipogenesis decreased from 25% to 17% in parallel to decreases in alanine aminotransferase and hepatic steatosis, consistent with the theory that de novo lipogenesis is a critical metabolic function linking dietary sugars and NAFLD.^67^

Unlike glucose, which is metabolized by most cells in the human body, fructose is metabolized primarily by the liver.^68^ Fructose metabolism is considered a major contributor to de novo lipogenesis in that it bypasses the rate-limiting enzyme of glycolysis (phosphofructokinase-1) and is rapidly phosphorylated by fructokinase C (the principal isoform of fructokinase in the liver) to fructose-1-phosphate without any negative feedback control.^69^ For this reason, fructose is considered more lipogenic than glucose. This reaction also requires ATP consumption, producing a drop in intracellular phosphate, ultimately resulting in purine nucleotide turnover and elevated uric acid formation (Figure 2).^70,71^ The decrease in ATP can induce oxidative stress and mitochondrial dysfunction.^72^ A study by Lanaspa et al demonstrated that the generation of mitochondrial oxidative stress from uric acid induces hepatic steatosis in HepG2 cells exposed to fructose.^72^ Furthermore, Choi et al confirmed that uric acid has direct effects on hepatic steatosis through the induction of endoplasmic reticulum stress and activation of SREBP-1c. SREBP-1c stimulates lipogenesis through the activation of lipogenic enzymes expressed in the liver, including acetyl-CoA carboxylase 1, fatty acid synthase, and stearoyl-CoA desaturase-1.^32^

The fatty acids accumulating in the liver can be esterified to form triglycerides and subsequently stored as lipid droplets or exported as VLDL particles into circulation.^73^ The storage of fatty acids in lipid droplets is thought to play a protective role in the disease process^74^ since excess fatty acid buildup can lead to the production of lipotoxic intermediates such as diacylglycerols, lysophosphatidylcholine species, ceramides, free cholesterol, and bile acids.^75^ These hepatotoxic lipids promote endoplasmic reticulum stress, mitochondrial dysfunction, and activation of NADPH oxidase, leading to downstream production of reactive oxygen species.^57,76^ These processes drive hepatic IR and inflammation, promoting disease progression to NASH and fibrosis (Figure 2). Additionally, hepatic glucotoxicity, which refers to the toxic effects of excess sugar intake on the liver, and hyperglycemia can disrupt insulin signaling.^75^ Hepatic IR enhances gluconeogenesis and represses insulin-dependent glycogen synthesis, leading to a vicious cycle of hyperglycemia and dysmetabolism, eventually causing hepatocyte injury and death.^75,77,78^

Furthermore, the secretion of triglyceride-enriched VLDL from the liver is increased in absolute terms in NAFLD; however, this increase does not match the overwhelming accumulation of lipids in the hepatocytes.^79^ The structure of VLDL comprises a triglyceride-enriched core with a monolayer of phospholipids that integrate proteins, such as apolipoprotein B-100, which are required for proper delivery and uptake of lipids to tissues. Impaired synthesis of apolipoprotein B-100 has been demonstrated in subjects with NASH, which may be related to disturbed redox balance and hyperinsulinemia.^80,81^ These processes lead to reduced VLDL assembly and excretion, resulting in hepatic lipid accumulation.^57^

### Sugars and extrahepatic mechanisms

Overconsumption of added sugars can cause adaptations beyond the liver in the brain, adipose tissue, skeletal muscle, and pancreas. These extrahepatic and central responses to high sugar intake produce downstream changes that can indirectly contribute to hepatic steatosis. Added sugar intake triggers neuroadaptations in the mesolimbic reward pathway, promoting hedonic caloric intake above energy needs, leading to increased adiposity.^82^ Appetite and satiety levels are influenced by the type of sugar consumed. Glucose intake stimulates insulin secretion from the pancreatic beta cells, whereas fructose has a negligible impact on circulating insulin levels. Leptin (the satiety hormone) is triggered by insulin-mediated glucose metabolism, while fructose bypasses leptin secretion and attenuates the suppression of ghrelin (the hunger hormone), increasing appetite.^83^ Shapiro et al demonstrated that high fructose intake also induced leptin resistance in rats.^84^ These changes can eventually lead to obesity and peripheral insulin resistance, promoting hepatic steatosis since hyperinsulinemia triggers adipose tissue lipolysis, causing an increase in free fatty acid delivery to the liver.^57^ Additionally, insulin resistance in skeletal muscle can promote increased hepatic de novo lipogenesis by diverting ingested glucose away from muscle glycogen synthesis toward the liver for hepatic triglyceride synthesis.^85–87^

### Sugars and genetic mechanisms

While the addition of high levels of simple sugars to the diet is a relatively modern phenomenon, the genetic underpinnings driving biological responses to this diet variant are not. There are a number of genetic polymorphisms linked to NAFLD in humans, but one of the most common appears to further explain the sugar–NAFLD relationship. The single nucleotide polymorphism in the PNPLA3 gene (I148 M variant, rs738409), which encodes a 481 amino acid protein called adiponutrin, is strongly associated with NAFLD in the setting of increased insulin resistance and body mass index.^88,89^ This polymorphism promotes hepatic steatosis by inhibiting the activity of lipases on the surface of lipid droplets, reducing triglyceride mobilization.^90^ Individuals who carry this variant are more susceptible to increased hepatic fat accumulation when sugar consumption is high.^91^ In the United States, the highest frequency of PNPLA3 rs738409 was found among those of Latino/Hispanic origin (0.49) compared to European ancestry (0.23) and African ancestry (0.17).^88^ A nutrigenetic analysis in Latino/Hispanic children demonstrated that triglyceride accumulation in the liver was dependent on sugar intake in those with the homozygous GG substitution of the PNPLA3 genotype.^91^ This evidence suggests that added sugar consumption may exacerbate the effects of this polymorphism by increasing intrahepatic lipid volume in the setting of a decreased ability to mobilize and remove lipids. Little is known about the impact of dietary sugars on other genes, including the glucokinase regulatory gene, which regulates de novo lipogenesis by controlling hepatocyte glucose influx, and the transmembrane 6-superfamily member 2, which regulates VLDL secretion.^92^ Therefore, more research is needed to investigate the interaction between dietary sugars and these genetic polymorphisms in NAFLD pathogenesis.^13^

## THE BURDEN OF DIETARY SUGARS

Globally, the food industry in high-income countries has become saturated with appetizing and potentially addictive products containing added sugars.^93,94^ This movement is now increasingly apparent in low-income and middle-income countries.^95^ In food production, sugar is not only used as a caloric sweetener but also as a bulking and browning agent, humectant, texture modifier, fermentation substrate, and preservative.^96^ As a result, added sugars are a ubiquitous component of processed foods and beverages in the food supply.^97^ With the myriad of synonyms for sugar used on nutrition labels, manufacturers disguise the total sugar content in food products, making efforts to reduce consumption challenging.^98^

### The biology of sugar preferences

Human sensory systems have evolved to detect and prefer the once rare, calorie-rich, sweet-tasting foods.^99,100^ When food availability was scarce, sweet foods were likely a vital energy source. Unfortunately, this biological preference makes humans vulnerable to today’s food environment, which is abundant in processed foods rich in refined sugars.^101^ Fructose is particularly problematic since it is the sweetest tasting natural sugar, 1.2–2.0 times sweeter than table sugar.^102^ The consumption of fructose has increased by 30% in the last 40 years and by 500% over the last century due to the increased consumption of processed foods and SSB.^103^

Recent studies in animal models and humans have shown that added sugars have habit-forming properties similar to alcohol, tobacco, cocaine, and caffeine.^104,105^ Excessive consumption of sugars can stimulate the reward pathways in the brain through an intense dopamine release.^82^ According to DiNicolantonio et al, chronic sugar intake can lead to “dopamine deficiency” in the brain due to the downregulation of the dopamine D2 receptors. Over time, this can lead to withdrawals, promoting the drive for perpetual sugar intake and addiction.^106^

People of all ages are susceptible to the pervasiveness of added sugar. Today, an American child as early as 2 years of age is more likely to consume a sugar-sweetened product than a fruit or vegetable.^107^ Given that food preferences are established in early childhood, this may drive lifelong diet preferences.^108^ The distinction between fruits, which naturally contain glucose and fructose, and processed foods with added sugar should be noted. Despite their sweet taste, fruits contain relatively low amounts of simple sugars compared to processed foods and SSB. Additionally, whole fruits contain fiber and antioxidants, which may protect against the harmful metabolic effects of sugar consumption.^109^

### Hepatic consequences of added sugars

Excessive added sugar intake, especially fructose, is linked with metabolic abnormalities that can cause NAFLD, which can progress to advanced liver disease.^110,111^ In children and adolescents, fructose has a dose-dependent correlation with NAFLD onset and development of fibrosis.^13^ A recent meta-analysis revealed a significant positive association between higher consumption of SSB and the risk of NAFLD.^112^ According to Geidl-Flueck et al, even modest consumption of added sugars from beverages sweetened with fructose or sucrose over several weeks led to increased hepatic fatty acid synthesis in healthy men.^113^ Additionally, a systematic review of 7 studies including 4639 subjects showed that SSB consumers had a 53% increased risk of developing NAFLD compared with nonconsumers.^114^ In a group of otherwise healthy, overweight adults, the consumption of SSB for 6 months significantly increased hepatic steatosis, skeletal muscle fat, and visceral fat.^115^ This suggests that the daily consumption of SSB leads to an increased risk of metabolic and CVDs, including NAFLD. While earlier human studies are confounded by the fact that fructose was provided in the context of overfeeding and weight gain, more recent studies show that the effects of dietary fructose are independent of caloric intake and body mass index.^116–118^

### Extrahepatic consequences of added sugar

With excessive sugar consumption, NAFLD typically occurs in the setting of obesity, dysglycemia, atherogenic dyslipidemia, and hypertension.^8^ Eventually, extrahepatic manifestations of NAFLD can occur, including chronic kidney disease and CVD,^119^ the leading cause of death among NAFLD patients.^120^ Additionally, NAFLD may play a role in the onset and progression of T2D and metabolic syndrome rather than just being an outcome of these conditions.^121,122^ A recent meta-analysis revealed that NAFLD was associated with about a 2-fold increase in the risk of incident T2D and metabolic syndrome over a median follow-up of 5 years.^123^

## HOPE FOR THE FUTURE: THE BENEFITS OF SUGAR REDUCTION FOR NAFLD

Lifestyle change is the first-line therapy for NAFLD.^124^ To prevent the development of NAFLD and its complications, it is imperative for our society to adopt interventions to support sugar reduction in the population, especially in children who are most vulnerable.^125^ Potential strategies include food education, the distribution of fruits and vegetables at school, and increased taxes on foods that contain any form of added sugar.^125^

### Taxation

Sugar taxation has recently emerged as a viable strategy in many countries (eg, Mexico, Denmark, and South Africa) and US jurisdictions (eg, Berkeley, California).^126–129^ A recent systematic review revealed that sugar taxation is a cost-effective policy option to reduce the health and economic burden related to the overconsumption of added sugars. These savings were driven by avoided healthcare costs and tax revenue exceeding intervention costs.^130^ Additionally, a microsimulation model developed by Vreman et al assessed the health and economic benefits of interventions aimed at reducing the intake of added sugars and estimated that a 20% reduction in added sugar intake would significantly reduce the prevalence of hepatic steatosis, NASH, cirrhosis, HCC, obesity, T2D, and CVD. Direct medical costs and disease-attributable disability-adjusted life years would also be reduced; these effects increased proportionally when added sugar intake was reduced by 50%.^131^

### Individual interventions

Clinical trials have demonstrated that dietary sugar restriction interventions that include meal provision and instruction are particularly effective therapeutic strategies for NAFLD. One short-term intervention study in 41 children (9–18 y) with obesity showed that dietary restriction of the free sugar fructose (~4% of total energy intake) via meal provision was associated with improvements in hepatic steatosis (from 7.2% to 3.8%), hepatic de novo lipogenesis, and insulin kinetics.^35^ Aligning with this, Schwimmer et al, recently conducted an 8-week, randomized, controlled intervention study in 40 adolescent boys with NAFLD (11–16 y.) to test the effect of dietary free sugar restriction on hepatic fat and found that the diet treatment group achieved a significantly greater reduction in hepatic fat (from 25% at baseline to 17% at week eight) compared to the usual control diet (21%–20%).^33^ In this study, meals were provided for the whole family along with individualized menu planning to restrict free sugars to less than 3% of total energy needs in the intervention diet group. A subsequent analysis of the same study participants by Cohen et al evaluated the effects of the 8-week sugar restriction on hepatic de novo lipogenesis, measured as the percentage contribution to plasma triglyceride palmitate using a 7-day metabolic labeling protocol with heavy water. Results revealed that the dietary sugar restriction significantly reduced hepatic de novo lipogenesis and fasting insulin among adolescents with NAFLD.^67^ Furthermore, plasma metabolomics and metagenomics were performed to investigate the systemic changes that occurred with the reduction in steatosis and de novo lipogenesis. The analyses revealed that the low free sugar diet, compared to the usual diet, was associated with metabolome and microbiome changes that may reflect biological mechanisms linking dietary sugar restriction to a therapeutic decrease in hepatic fat.^132^

On the other hand, a recent study by Schmidt et al, found that a dietitian-led sugar reduction intervention did not improve liver outcomes in Latino youth with obesity (11–18 y of age).^133^ However, this study only involved nutrition education without meal provision, and the goal for sugar restriction was only ≤ 10% of total calories.

The combination of a low-sugar diet and time-restricted feeding (16 consecutive hours of fasting and 8 hours of eating time daily) has also been shown to reduce adiposity and improve markers of liver function, dyslipidemia, and inflammation in adult patients with NAFLD.^134^ Another study in overweight or obese adults with NAFLD showed that a low free sugar diet intervention over 12 weeks resulted in reduced fibrosis scores and steatosis scores with improved glycemic indices and decreased concentrations of biomarkers of inflammation, triglycerides, and total cholesterol levels.^135^ Moreover, a study in children and adolescents (7–18 y.) with NAFLD found that a low fructose and low glycemic index/load dietary intervention over 6 months resulted in improvements in body composition and plasma markers of liver dysfunction and cardiometabolic risk.^136^

Further research is needed to confirm the long-term effectiveness of sugar reduction for NAFLD prevention and treatment, along with continued public health initiatives to decrease added sugars in our food system. Please see Table 1 for a detailed summary of the studies focusing on taxation and sugar restriction mentioned above.

**TABLE 1:** Sugar taxation & restriction studies

Author, Year	Study population	Study design	Intervention	Main findings
Sugar taxation studies with SSB consumption–focused outcomes				
Colchero et al, 2016^126^	6253 households in 53 cities in Mexico	Observational study using data from Nielsen Mexico’s Consumer Panel Services on the purchase of beverages in Mexico from January 2012 to December 2014	Excise tax of 1 peso/L on SSB	Purchase of taxed beverages decreased by an average of 6% and at an increasing rate of up to a 12% decline by December 2014. Reductions are higher among households of low SES.
Falbe et al, 2016^127^	Low-income neighborhoods in Berkeley vs. the comparison cities of Oakland and San Francisco, California	Repeated cross-sectional study to examine changes in pre-tax to post-tax beverage consumption based on data from an interviewer-administered beverage frequency questionnaire	Excise tax ($0.01/oz) on SSB	Consumption of SSBs decreased by 21% in Berkeley and increased by 4% in comparison cities (*P* = .046). Water consumption increased more in Berkeley (+ 63%) than in comparison cities (+ 19%; *P* < 0.01).
Sugar restriction studies with liver health–focused outcomes				
Schwarz et al, 2017^116^	41 nondiabetic Latino and African American children (9–18 y) with obesity and metabolic syndrome, who identified as having habitual high sugar consumption (fructose intake >50 g/d)	Convenience cohort within-subject intervention with repeated measures	Meal provision restricting fructose ingestion only to naturally occurring fructose in fruits and vegetables (~ 15 gm/d/ 4% of total kcal, for 9 d) by substituting complex carbohydrates for excess dietary fructose while maintaining a neutral energy balance	Liver fat decreased from a median of 7.2% to 3.8% (*P* < .001). VAT decreased from 123 cm^3^ to 110 cm^3^ (*P* < .001). The DNL AUC decreased from 68% to 26% (*P* < 0.001). Insulin kinetics improved (*P* < 0.001). Changes occurred irrespective of baseline liver fat.
Schwimmer et al, 2019^33^	40 adolescent boys with biopsy-confirmed NAFLD, ages 11–16 y	Randomized, parallel assignment clinical trial without blinding	8-week LFSD: Individualized menu planning and meal provision for the entire household to restrict free sugar intake to less than 3% of daily calories	Mean decrease in hepatic steatosis from baseline to week 8 was significantly greater for the intervention diet group (25%–17%) vs. the usual diet group (21%–20%), and the adjusted week 8 mean difference was −6.23% (*P* < 0.001).
Cohen et al., 2021^67^	40 adolescent boys with biopsy-confirmed NAFLD, ages 11–16 y	Randomized, parallel assignment clinical trial without blinding	8-week LFSD (see Schwimmer et al., 2019, 2019 above) + 7-day metabolic labeling protocol with heavy water	Hepatic DNL was significantly decreased in the treatment group (from 34.6% to 24.1%) vs. the control group (33.9%–34.6%), along with greater decreases in hepatic fat and fasting insulin.
Cohen et al., 2023^132^	40 adolescent boys with biopsy-confirmed NAFLD, ages 11–16 y	Randomized, parallel assignment clinical trial without blinding	8-week LFSD (see Schwimmer et al., 2019 above) + plasma metabolomics analysis	The LFSD treatment, compared to the usual diet, was associated with differential expression of 419 metabolite features (*P* < 0.05), which were enriched in amino acid pathways, including methionine/cysteine and serine/glycine/alanine metabolism (*P* < 0.05), and lipid pathways, including omega-3 and linoleate metabolism (*P* < 0.05).Microbiome changes included an increase in richness at the phylum level and changes in a few genera within *Firmicutes*.
Schmidt et al, 2022^133^	105 Latino adolescents (11–18 y) with obesity (BMI ≥95th percentile for age & sex)	Parallel-design randomized controlled dietary intervention trial	A 12-week dietitian-led sugar reduction intervention, including nutrition education to promote free sugar reduction to ≤ 10% of total calorie needs	Mean free sugar intake decreased in the intervention group vs. control (11.5%–7.3% compared with 13.9%–10.7% of total energy needs, respectively; *P* = 0.02). There were no significant effects on liver outcomes or anthropometrics *(P* all > 0.10).
Kord-Varkaneh et al, 2023^134^	52 overweight/obese adults with NAFLD, ages 18–50 y	Randomized clinical trial	A 12-week time-restricted feeding intervention (16 h fasting/8 h feeding daily) + a low-sugar diet (< 3% total energy needs)	The time-restricted feeding intervention group reduced body fat, body weight, WC, BMI, fasting blood glucose, liver enzymes (ALT, AST, GGT), lipids (TG, TC, LDL-cholesterol), and inflammatory markers (hs-CRP and cytokeratin-18), all statistically significant vs. control (*P*<0.05).
Khodami et al, 2022^134^	43 overweight/obese adults with FibroScan-proven NAFLD, ages 18–60 y	Randomized two-arm, parallel dietary intervention	12-week LFSD intervention (dietitian instruction to limit free sugars to <10% of total energy needs)	The LFSD intervention group compared with the usual diet control group, significantly decreased ALT, TG, TC, FBS, insulin, HOMA-IR, hs-CRP, TNF-α, and NF-kb (*P* < 0.05). The LFSD group also reduced fibrosis score and steatosis score, with increased QUICKI compared to the control (*P* < 0.05).
Mager et al., 2015^136^	Children and adolescents with NAFLD (n = 12) and healthy controls (n = 14), ages 7–18 y	Prospective dietary intervention	Dietary education/sample menus to promote the consumption of a low fructose (< 7% energy needs) and low glycemic index (45–55)/glycemic load (< 80) (FRAGILE) diet over 6 mo	In children with NAFLD, there were significant reductions in SBP, percentage BF, and plasma concentrations of ALT (*P* = 0.04), Apo-B-100 (*P* < .001), and HOMA-IR at 3 and 6 mo (*P* < 0.05). Dietary reductions in fructose and GL were related to reductions in SBP (*P* = 0.01), ALT (*P* = 0.004), HOMA-IR (*P* = 0.03), and percentage BF. No changes in laboratory variables were observed in the healthy control group except for Apo-B-100.

Abbreviations: ALT, alanine aminotransferase; Apo-B-100, apolipoprotein B-100; AST, aspartate aminotransferase; BF, body fat; BMI, body mass index; DNL, de novo lipogenesis; FBS, fasting blood sugar; GGT, gamma-glutamyl transferase; GL, glycemic load; HOMA-IR, Homeostatic Model Assessment for Insulin Resistance; hs-CRP, high sensitivity C-reactive protein, TG, triglycerides; LFSD, low free sugar diet; QUICKI, quantitative insulin sensitivity check index; SBP, systolic blood pressure; SSB, sugar-sweetened beverages; TC, total cholesterol; VAT, visceral adipose tissue; WC, waist circumference.

## SUGAR REDUCTION AND PRECISION NUTRITION

Dietary recommendations for obesity issued over the past decade have aimed to mitigate disease progression using a one-size-fits-all approach.^137,138^ This strategy has shown only moderate success, and some recommended strategies for generalized weight loss may not be the most effective in the setting of elevated hepatic fat and alanine aminotransferase.^139–145^ Recent studies have demonstrated that personalized nutrition interventions tailored to individuals or subgroups of individuals are more effective than conventional dietary advice.^146^ Precision nutrition (aka personalized nutrition) utilizes information on individual characteristics or phenotypes of disease to develop targeted nutritional recommendations.^147,148^

NAFLD is a heterogeneous condition with a broad spectrum of clinical manifestations, pathogenesis, and response to treatment.^149–151^ This heterogeneity is thought to arise from multiple factors, including sex, hormonal status, genetics, gut microbiota composition, other comorbidities, and certain exposures, including diet and physical activity.^150^ Carrillo-Larco et al identified 3 phenotypes in adults with NAFLD using a machine-learning approach. The majority of subjects fell into the average NAFLD phenotype, whereas 6% and 10% of the remaining subjects fell into phenotypes characterized by (1) high levels of anthropometrics, systolic blood pressure, and glucose (highest all-cause mortality), or (2) high levels of liver biomarkers and cholesterol, respectively.^152^ Furthermore, recent studies have demonstrated that diet and lifestyle modifications, including sugar reduction, are more effective in decreasing intrahepatic fat in NAFLD patients who are carriers of the PNPLA3 I148M gene polymorphism versus noncarriers.^153,154^ A tailored dietary approach that accounts for differences in clinical, genetic, and metabolic characteristics between responders and nonresponders to nutrition therapies for NAFLD, including sugar reduction, may be more effective than traditional lifestyle recommendations. Indeed, Zelber-Sagi et al suggested that current dietary regimens for NAFLD, such as low carbohydrate or reduced refined sugar interventions, could be improved by tailoring nutrition recommendations to meet individual preferences and goals, therefore improving long-term adherence and health outcomes.^138^ Although precision nutrition interventions have shown promise in several chronic disease populations,^147,155^ there is limited research in patients with NAFLD. Further research is needed to determine subphenotypes of NAFLD and tailor nutrition advice to individuals or subgroups of similar individuals with NAFLD to improve health outcomes.

## CONCLUSION

In conclusion, NAFLD is the most common cause of chronic liver disease and is strongly associated with the metabolic conditions of insulin resistance, T2D, and obesity. The increasing prevalence of NAFLD is linked to the rise of sugar consumption; therefore, dietary strategies incorporating restriction may provide an effective disease prevention and treatment solution. While the role of dietary sugar in NAFLD pathogenesis is still being elucidated, current consumption levels surpass the World Health Organization's guideline of no more than 10% of total energy intake. Given the health and economic impact of NAFLD, it's crucial to reduce free sugar intake to alleviate the current burden and prevent future obesity-related comorbidities.
